# High zoonotic potential of *Cryptosporidium* spp., *Giardia duodenalis*, and *Enterocytozoon bieneusi* in wild nonhuman primates from Yunnan Province, China

**DOI:** 10.1186/s13071-022-05217-7

**Published:** 2022-03-12

**Authors:** Fanfan Shu, Shujiao Song, Yanting Wei, Falei Li, Yaqiong Guo, Yaoyu Feng, Lihua Xiao, Na Li

**Affiliations:** 1 Center for Emerging and Zoonotic Disease, College of Veterinary Medicine, South China Agricultural University, Guangzhou, 510642 Guangdong China; 2 Guangdong Laboratory for Lingnan Modern Agriculture, Guangzhou, 510642 Guangdong China; 3 Key Laboratory of Veterinary Public Health of Yunnan Province, College of Veterinary Medicine, Yunnan Agricultural University, Kunming, 650201 Yunnan China

**Keywords:** *Cryptosporidium*, *Giardia duodenalis*, *Enterocytozoon bieneusi*, Nonhuman primates, Zoonotic potential

## Abstract

**Background:**

*Cryptosporidium* spp., *Giardia duodenalis* and *Enterocytozoon bieneusi* are important zoonotic protists in humans and animals around the world, including nonhuman primates (NHPs). However, the prevalence, genetic identity and zoonotic potential of these pathogens in wild NHPs remain largely unclear.

**Methods:**

A total of 348 fecal samples were collected from wild NHPs at four locations in Yunnan, southwestern China, and analyzed for these pathogens using nested PCR targeting various genetic loci and DNA sequence analysis of the PCR products. The zoonotic potential of the pathogens was assessed by comparing the genetic identity of the pathogens in these animals with that previously reported in humans.

**Results:**

Altogether, two (0.6%), 25 (7.2%) and 30 (8.6%) samples were positive for *Cryptosporidium* sp., *G. duodenalis* and *E. bieneusi*, respectively. The *Cryptosporidium* sp. identified belonged to *C. parvum* subtype IIdA20G1. Both assemblages A (*n* = 3) and B (*n* = 22) were identified among *G. duodenalis*-positive animals. Five genotypes in zoonotic Group 1 were identified within *E. bieneusi*, including Type IV (*n* = 13), D (*n* = 7), Peru8 (*n* = 6), MMR86 (*n* = 2) and HNFS01 (*n* = 2). All genotypes and subtypes identified are known human pathogens or phylogenetically related to them.

**Conclusions:**

Data from this study suggest a common occurrence of zoonotic genotypes of *G. duodenalis* and *E. bieneusi* in wild NHPs in southwestern China.

**Graphical Abstract:**

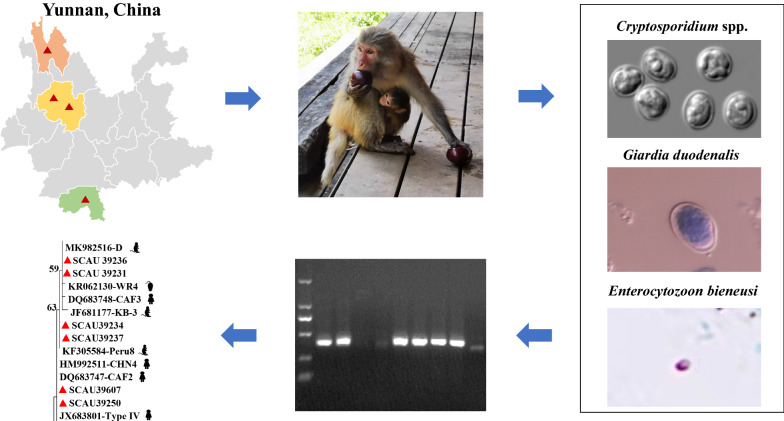

## Background

Cryptosporidiosis, giardiasis and microsporidiosis are three emerging zoonotic diseases caused by *Cryptosporidium* spp., *Giardia duodenalis*, and *Enterocytozoon bieneusi*, respectively, that have been reported in humans as well as domestic and wild animals. Humans can be infected with these pathogens through the ingestion of contaminated food or water, causing diarrhea in immunocompetent individuals and significant mortality in immunocompromised ones [1–3]. To date, over 40 *Cryptosporidium* species and 100 genotypes have been recognized, including *Cryptosporidium parvum*, which has a broad host range [4]. Similarly, at least eight distinct assemblages (i.e., A–H) are known in *G. duodenalis* [5]. Among these, assemblages A and B have a broad host range and as such can be transmitted between humans and animals, whereas assemblages C–H are host specific [6]. For *E. bieneusi*, the nearly 500 genotypes identified thus far have been classified into 11 major groups, with Group 1 genotypes having a broad host range [7].

Nonhuman primates (NHPs), due to their high genetic homology to humans, are considered potential sources of zoonotic parasites in humans [8]. Data from several studies indicate that captive and farmed NHPs are reservoirs of *Cryptosporidium* spp., *G. duodenalis* and *E. bieneusi*, with the most prevalent genotypes of the pathogens also detected in humans [9]. Compared with captive NHPs in zoos or breeding facilities, free-range NHPs are in more contact with humans and can thus potentially transmit pathogens to humans [9].

Yunnan Province in China is home to many wild NHPs [10]. The high biodiversity and lifestyle of native ethnic groups facilitate the contact of people with wild animals [11]. For example, in Yunnan Golden Monkey National Park in northern Yunnan, snub-nosed monkeys (*Rhinopithecus bieti*) are in frequent contact with local Tibetan residents [12]. In the Jizu Mountains and Shibao Mountains of western Yunnan, rhesus macaques (*Macaca mulatta*) are in close contact with monks living in temples and with pilgrims and tourists visiting the mountains [13]. In Xishuangbanna Primeval Forest Park, which is a famous tropical rainforest reserve in southern Yunnan, tourists are encouraged to feed and interact with NHPs [13].

In a small-scale survey of animals, captive NHPs from zoos and research laboratories in Yunnan Province were shown to be infected with several genotypes of *E. bieneusi* [14]. A few studies on the genetic identity of *Cryptosporidium* spp., *G. duodenalis* and *E. bieneusi* in NHPs have also been conducted in China with captive animals [8, 9, 15–21]. There is a need for molecular characterizations of these pathogens in wild NHPs. In the study presented here, four tourist attractions in Yunnan Province home to many wild NHPs were examined for the prevalence, genetic identity and public health potential of *Cryptosporidium* spp., *G. duodenalis* and *E. bieneusi* in these animals.

## Methods

### Sample collection

**Fig. 1:**
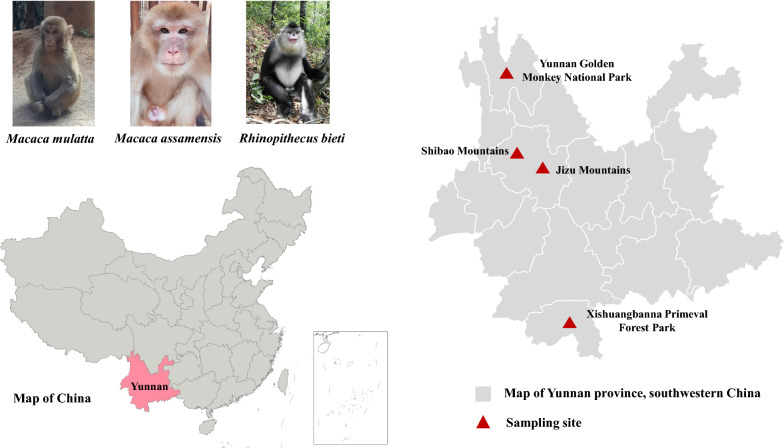
Sampling sites for enteric protists in nonhuman primates in Yunnan Province, China

A total of 348 fecal samples from rhesus macaques (*M. mulatta*, *n* = 320), snub-nosed monkeys (*R. bieti*, *n* = 20), and Assamese macaques (*Macaca assamensis*, *n* = 8) at four tourist attractions in Yunnan Province, China were collected from June 2019 to January 2021 (Table 1; Fig. 1). As there are only a limited number of Assamese macaques in Xishuangbanna Primeval Forest Park, to better protect them, they are kept in an isolated area that is separate from those frequented by rhesus macaques. The sampling size was strictly controlled not to exceed one third of the estimated total number of NHPs at each sampling location. Each sample was from one animal and consisted of a fresh fecal dropping collected from the ground. All monkeys appeared to be clinically normal without obvious signs of diarrhea. The samples were stored at 4 °C in 2.5% potassium dichromate prior to DNA extraction and PCR analysis.

**Table 1:** Description of fecal samples collected for analysis from three species of nonhuman primates at four locations in Yunnan Province, China

Location	Jurisdiction	Geographical coordinates	Altitude (m)	No. of samples			Total (*n*)
				*Macaca mulatta*	*Macaca assamensis*	*Rhinopithecus bieti*	
Yunnan Golden Monkey National Park	Diqing Tibetan Autonomous Region	99°37′E, 27°61′N	2800–3100	0	0	20	20
Jizu Mountains	Dali Bai Autonomous Region	100°41′E, 25°95′N	1800–1861	71	0	0	71
Shibao Mountains	Dali Bai Autonomous Region	99°84′E, 26°39′N	1900–2000	116	0	0	116
Xishuangbanna Primeval Forest Park	Xishuangbanna Dai Autonomous Region	100°84′E, 21°93′N	720–1355	133	8	0	141
Total	–	–	–	320	8	20	348

### DNA extraction and PCR

Before genomic DNA extraction, approximately 300 mg of fecal material was washed with distilled water by centrifugation (2000 *g*, 10 min). DNA was extracted from the pellet using a FastDNA Spin Kit for Soil (MP Biomedicals, Solon, OH, USA) as previously described [22]. The extracted DNA was stored at – 20 °C until being analyzed by PCR.

### PCR amplification

*Cryptosporidium* spp. were detected and genotyped by PCR and sequence analyses of the small subunit (*SSU*) rRNA gene [23]. The *C. parvum* detected was further subtyped by sequence analysis of the 60-kDa glycoprotein (*gp*60) gene [24]. In contrast, *G. duodenalis* was detected and genotyped by PCR and sequence analyses of the β-giardin (*bg*), triosephosphate isomerase (*tpi*) and glutamate dehydrogenase (*gdh*) genes [25–27]. *Enterocytozoon bieneusi* in the extracted DNA was detected and genotyped by PCR and sequence analyses of a rRNA fragment containing the entire internal transcribed spacer (ITS) [28]. Each sample was subjected to PCR analysis with two technical replicates at each genetic locus. DNA preparations of *Cryptosporidium tyzzeri* from mice, assemblage G from mice and genotype PtEb IX from dogs were used as positive controls in the PCR analysis for *Cryptosporidium* spp., *G. duodenalis* and *E. bieneusi*, respectively, whereas reagent-grade water was used as the negative control.

### Sequence analysis

All positive secondary PCR products were sequenced by Sangon Biotech (Shanghai, China) bidirectionally on an ABI 3730 Genetic Analyzer (Applied Biosystems, Thermo Fisher Scientific, Foster City, CA, USA). The raw sequences obtained were assembled using ChromasPro 1.33 (http://technelysium.com.au/ChromasPro.html), edited using BioEdit 7.1 (http://www.mbio.ncsu.edu/BioEdit/bioedit.html) and aligned with reference sequences from GenBank using ClustalX 2.1 (http://clustal.org). Genotypes and subtypes of the pathogens were named according to the established nomenclature [29, 30]. A maximum likelihood (ML) tree was constructed to evaluate the phylogenetic relationships among genotypes of *E. bieneusi* using MEGA 7.0.14 (http://www.megasoftware.net/) with substitution rates calculated with the general time-reversible model. The reliability of the tree was assessed using bootstrap analysis with 1000 replicates.

### Statistical analysis

The Chi-square test was used to compare differences in infection rates of pathogens between geographical locations. Differences were considered to be significant at *P* < 0.05.

## Results

### Occurrence and genetic identity of *Cryptosporidium* sp.

Of the 348 fecal samples analyzed, only two (0.6%) samples from rhesus macaques in Xishuangbanna Primeval Forest Park were positive for *Cryptosporidium* (Table 2). Sequence analysis of the *SSU* rRNA PCR products identified both as *C. parvum*. One sample, SCAU39270, was successfully subtyped at the *gp60* locus as IIdA20G1, while the expected *gp*60 PCR product in the other *C. parvum*-positive sample (SCAU39236) could not be generated.

**Table 2:** Occurrence and identity of enteric protists in nonhuman primates in Yunnan Province, China by sampling location and animal species

Variables	No. of specimens	*Cryptosporidium parvum*		*Giardia duodenalis*		*Enterocytozoon bieneusi*	
		No. positive (%)	Subtype (*n*)	No. positive (%)	Genotype (*n*)	No. positive (%)	Genotype (*n*)
Location							
Yunnan Golden Monkey National Park	20	0 (0)	–	2 (10.0)	B (2)	0 (0)	–
Jizu mountains	71	0 (0)	-	11 (15.5)^a^	B (9), A (2)	4 (5.6)	MMR86 (2), HNFS01 (2)
Shibao mountains	116	0 (0)	-	8 (6.9)	B (7), A (1)	2 (1.7)	Type IV (1), D (1)
Xishuangbanna Primeval Forest Park	141	2 (1.4)	IIdA20G1(1)	4 (2.8)	B (4)	24 (17.0)^b,c^	Type IV (12), Peru8 (6), D (6)
Animal species							
*Macaca mulatta*	320	2 (0.6)	IIdA20G1(1)	23 (7.2)	B (20), A (3)	27 (8.4)	Type IV (13), Peru8 (5), D (5), MMR86 (2), HNFS01 (2)
*Macaca assamensis*	8	0 (0)	-	0 (0)	-	3 (37.5)	D (2), Peru8 (1)
*Rhinopithecus bieti*	20	0 (0)	-	2 (10.0)	B (2)	0 (0)	-
Total	348	2 (0.6)	IIdA20G1(1)	25 (7.2)	B (22), A (3)	30 (8.6)	Type IV (13), D (7), Peru8 (6), MMR86 (2), HNFS01 (2)

^a^*P* = 0.0006, for Jizu Mountains in comparison with Xishuangbanna Primeval Forest Park

^b^*P* = 0.0208, for Xishuangbanna Primeval Forest Park in comparison with Jizu Mountains

^c^*P* = 0.00005, for Xishuangbanna Primeval Forest Park in comparison with Shibao Mountains

### Occurrence and assemblages of *G. duodenalis*

In nested PCR analyses of the three genetic loci, 25 (7.2%) of the 348 fecal samples were positive for *G. duodenalis*, with infection rates ranging from 2.8% (4/141) to 15.5% (11/71) among the four sampling locations (Table 2). Among these, the infection rate was significantly higher among samples collected at Jizu Mountains (15.5%) than among those collected at Xishuangbanna Primeval Forest Park (2.8%; *χ*^2^ = 11.504, *P* = 0.0006). DNA sequence analysis of the *bg*, *tpi* and *gdh* PCR products revealed the presence of assemblages A (*n* = 3) and B (*n* = 22) in those monkeys. By animal species, only *M. mulatta* and *R. bieti* samples were positive for *G. duodenalis* (Table 2), with *R. bieti* only positive for assemblage B and *M. mulatta* positive for both assemblages A and B. The assemblage A was identified as A1 and A5 subtypes at the *bg* and *tpi* loci and as A5 subtype at the *gdh* locus; thus, all belonged to the AI subassemblage.

### Occurrence and genotypes of *E. bieneusi*

**Fig. 2:**
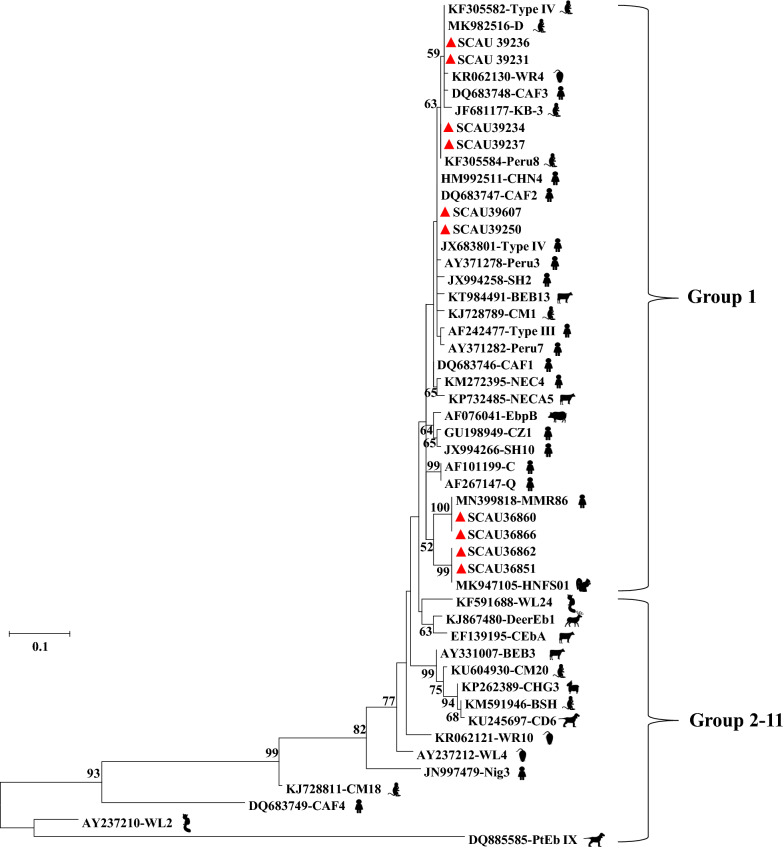
Phylogenetic relationship of *Enterocytozoon bieneusi* genotypes from wild nonhuman primates in Yunnan Province, China based on a maximum-likelihood analysis of sequences of the ribosomal internal transcribed spacer. Bootstrap values > 50% from 1000 replicate analysis are shown on nodes. Representative sequences obtained from this study are indicated with red triangles. The major hosts of the genotypes are indicated with black animal symbols

Of the 348 fecal samples collected from wild NHPs, 30 (8.6%) were positive for *E. bieneusi* in the PCR analysis of the ITS, with infection rates ranging from 1.7% (2/116) to 17.0% (24/141) among the four locations (Table 2). The infection rate at Xishuangbanna Primeval Forest Park (17.0%) was significantly higher than at Jizu Mountains (5.6%; *χ*^2^ = 5.341, *P* = 0.0208) and Shibao Mountains (1.7%; *χ*^2^ = 16.377, *P* = 0.00005). Five *E. bieneusi* genotypes were detected in these animals, including Type IV (*n* = 13), D (*n* = 7), Peru8 (*n* = 6), MMR86 (*n* = 2) and HNFS01 (*n* = 2). The sequences of Type IV, D, Peru8, MMR86 and HNFS01 were identical to GenBank sequences JX683801(from humans), MK982516 (from giraffes), KF305584 (from rhesus macaques), MN399818 (from humans) and MK947105 (from flying squirrels), respectively. In the phylogenetic analysis, all were placed in zoonotic Group 1 (Fig. 2).

By animal species, 8.4% (27/320) *M. mulatta* and 37.5% (3/8) *M. assamensis* sampled were positive for *E. bieneusi*. Among these, all five subtypes (Type IV, Peru8, D, MMR86 and HNFS01) were detected in *M. mulatta* and two subtypes (D and Peru8) were found in *M. assamensis* (Table 2). None of the 20 *R. bieti* samples examined were positive for *E. bieneusi*.

### Coinfection of enteric pathogens

Among five samples from *M. mulatta* at Xishuangbanna Primeval Forest Park, one *C. parvum*-positive sample had concurrence of *E. bieneusi* genotype D, two samples (one with Peru8 and another with Type IV) had concurrence of assemblage B of *G. duodenalis* and two samples had concurrence of two *E. bieneusi* genotypes based on inconsistent sequencing results of the two PCR replicates (one with both Type IV and Peru8 and another with both Peru8 and D).

## Discussion

This is the first report of the occurrence of *Cryptosporidium* sp., *G. duodenalis* and *E. bieneusi* in wild NHPs in Yunnan Province, China. These pathogens were identified in 0.6, 7.2 and 8.6% of fecal samples, respectively. The occurrence rates of these pathogens are lower than those found in captive, laboratory and zoo NHPs in previous investigations, which reported infection rates ranging from 9.1% to 46.7% for *Cryptosporidium* spp. [8, 15, 31], 8.5% to 32.3% for *G. duodenalis* [8, 19, 32–34] and 11.4% to 46.2% for *E. bieneusi* [8, 14, 18, 19, 21, 35–38]. This difference was expected as the congregation of susceptible animals in an enclosed environment could facilitate the transmission of enteric pathogens on farms and in captivity.

The zoonotic IIdA20G1 subtype of *C. parvum* is the only *Cryptosporidium* identified in the present study. Thus far, three *Cryptosporidium* species, *C. hominis, C. parvum* and *C. muris*, have been identified as common ones in NHPs in China [15, 39]. Unlike IIa subtypes that are prevalent in many industrialized countries, IId subtypes are the most common *C. parvum* in China [39]. A previous study also found the IIdA15G2R1 and IIdA19G1 subtypes in NHPs in China [15, 36] and, more recently, the IIdA20G1 subtype was detected in cattle and deer in several areas in China, causing an outbreak of cryptosporidiosis in pre-weaned calves [40–43]. Therefore, the occurrence of this emerging subtype in NHPs indicates an expansion of host range of this emerging subtype.

Similar to other studies in NHPs, the zoonotic assemblage B was identified as the dominant genotype of *G. duodenalis* in *M. mulatta* and *R. bieti* in this study [8, 19, 20, 44, 45]. Assemblage B is the most common pathogenic genotype in humans in both industrialized and developing countries [5, 44]. The assemblage A identified in NHPs in the present study appears to belong to AI subassemblage, which is common in animals but nevertheless has been found in humans [30]. Thus, wild NHPs could serve as potential reservoirs for zoonotic *G. duodenalis*.

The five *E. bieneusi* genotypes detected in NHPs in Yunnan Province all belong to Group 1, which contains most of the zoonotic genotypes of major public health concern [7]. Among these, Type IV, D and Peru8 are known human and NHP pathogens in many countries [19, 46–48]. To our knowledge, our study is the first to report genotypes MMR86 and HNFS01 in NHPs in China. MMR86 was initially seen in humans in Myanmar, which borders Yunnan province, sampled in the present study [49], while HNFS01 was recently identified in flying squirrels in Henan, China (GenBank accession number MK947105). Previous studies on *E. bieneusi* in captive and farmed NHPs also detected mostly Group 1 genotypes [14, 18, 21, 35, 38, 50].

## Conclusions

In summary, the results of the present study show a common occurrence of zoonotic *G. duodenalis* and *E. bieneusi* genotypes in wild NHPs, which in turn represents a significant public health concern as tourists are coming into increasing contact with NHPs. In response, public education programs on the infective potential of pathogens from NHPs and the utility of hygiene in disease prevention should be implemented to inform park personnel and tourists as part of the One Health approach to the prevention and control of *Cryptosporidium* spp., *G. duodenalis* and *E. bieneusi* infections.

## Data Availability

Representative nucleotide sequences generated in the study were deposited in GenBank under the accession numbers OM212450, OM212451, OM212347, OM212012-OM212021, OM221354-OM221360.

## Abbreviations

*bg*: β-Giardin gene
*gdh*: Glutamate dehydrogenase gene
*gp60*: 60 KDa glycoprotein gene
ITS: Internal transcribed spacer
NHPs: Nonhuman primates
*SSU* rRNA: Small subunit rRNA gene
*tpi*: Triosephosphate isomerase gene
